# The role of multiplex PCR test in identification of bacterial pathogens in lower respiratory tract infections

**DOI:** 10.12669/pjms.305.5098

**Published:** 2014

**Authors:** Ozlem Aydemir, Yusuf Aydemir, Mehmet Ozdemir

**Affiliations:** 1 Ozlem Aydemir, Department of Microbiology, Sakarya University, Training and Research Hospital, Sakarya, Turkey.; 2 Yusuf Aydemir, Department of Pulmonology, Faculty of Medicine, Sakarya University, Sakarya, Turkey.; 3 Mehmet Ozdemir, Faculty of Medicine, Department of Microbiology, Necmettin Erbakan University, Konya, Turkey.

**Keywords:** Bacterial etiology, Culture, Lower respiratory tract infection, PCR

## Abstract

***Objectives: ***Lower respiratory tract infection is one of the most important causes of morbidity and mortality. However establishing a microbial diagnosis for patients with lower respiratory tract infection is still challenging and is often achieved in only half of cases by conventional methods. This study was designed to compare the fast responsive PCR method with the culture method in lower respiratory tract infections and to evaluate the reliability of multiplex PCR method.

***Methods: ***One hundred ninety seven patients with the symptoms of acute lower respiratory tract infection, and diagnosed with community-acquired pneumonia, acute exacerbation of chronic obstructive pulmonary disease and exacerbations of bronchiectasis were included in the study. Both culture and PCR methods was performed for the isolation of most commonly seen bacteria, from sputum, nasopharyngeal swabs and bronchoalveolar lavage fluid samples.

***Results: ***While at least one bacterial isolation was determined in 62 (31.5%) of all patients with culture method, this number increased to 125 (63.5%) with multiplex PCR. The bacteria most commonly identified by PCR were *S. pneumoniae* (32%) and *H. influenzae* (31%). There was a significant difference between PCR and culture in terms of multi-factor detection rates (p<0.005). Multiple bacteria were detected in only two cases in cultures; however, multiple pathogens were detected in 47 cases with PCR.

***Conclusions: ***Conventional methods, such as culture and serology are not always adequate to detect the pathogens in lower respiratory tract. Real-time PCR assays proved highly sensitive and rapid. The prevalence of bacteria and multiple agent detected by real-time PCR compared with culture was substantially higher. Widespread use of PCR methods, by providing the immediate and appropriate ''agent specific antibiotic treatment'' of LRTI, will help reduce failure and contributes to a reduction in antibiotic resistance.

## INTRODUCTION

Lower respiratory tract infection (LRTI) is one of the important causes of morbidity and mortality in the world. According to the WHO, LRTI is the number one cause of infection-related deaths and is the third leading cause of all deaths.^1^ Precise identification of the etiologic agents and early initiation of appropriate antimicrobial therapy is very important in treatment. Delays of 4-8 hours in starting treatment have been shown to increase mortality.^2^

Nevertheless, conventional diagnostic methods are often insufficient for etiological diagnosis, and in half of these cases the causative pathogen cannot be determined.^3^^-^^7^ The use of multiplex polymerase chain reaction (PCR), which is reported to be a reliable molecular method for diagnosing lower respiratory tract infections, has been used increasingly in recent years. The prominent advantage of PCR method compared to culture is that, since PCR is based on replicating the DNA or RNA of very small amount of microorganisms, it does not require living organisms and therefore is not affected by the prior use of antibiotics. In addition, PCR is more sensitive for detection of multiple microorganisms and delivers fast results.^8^^-^^10^

In this study, our primary aim was to compare the fast responsive PCR method with the culture method in LRTI and to evaluate the reliability of multiplex PCR method. The second aim of our study was to identify the bacterial pathogens that are the most common causes of LRTI and to use these results as a guide for the selection of experimental antibiotic regimen.

## METHODS

***Study design and population: ***Our study included 197 consecutive patients with LTRI who had been admitted to the Department of Thoracic Diseases and Pediatrics at the Public and University Hospital in Konya, Turkey from September 2012 to March 2013, and who were treated either as outpatient or inpatient. Acute-onset cough, new or increased mucopurulent sputum, body temperature above 37.8°C or below 36°C, pleuritic chest pain, dyspnea, increased CRP and sedimentation rate, WBC above 10,000 or below 4000, and radiological findings in the form of infiltration or consolidation were the conditions required for the diagnosis of community-acquired pneumonia (CAP). Patients were diagnosed with chronic obstructive pulmonary disease (COPD) based on medical history, physical examination and pulmonary function tests. Moreover, the existence of the above described three criteria such as dyspnea, increase in sputum purulence and sputum volume were required in the diagnosis of acute exacerbation. In addition, the bronchiectasis diagnosis was based on appropriate history and physical examination findings and was confirmed by high-resolution computed tomography. Patients with a history of hospitalization and antibiotic use within the prior ten days and with accompanying immunosuppression, respiratory failure, malignancy, and congestive heart failure were not included in the study. All patients were informed about the study and signed informed consent was obtained. The study was approved by the Ethics Committee of Meram Medical Faculty. (2012/33)

***Culture: ***The BAL samples were taken by the same pulmonologist by using the fiberoptic bronchoscope (Fujinon, Japan) and were put into a sterile container. Nasopharyngeal swabs (NFS) were taken by accessing the nostril with the swab. (eSwap Liquid Amies. Copan, Italy). The sputum samples were taken into a sterile container and were immediately analyzed microscopicallyby gram staining. The samples that were observed to have less than 10 epithelial cells and more than 25 leukocytes in each area upon 100x magnification were included in the study as suitable sputum samples. After gram staining and direct microscopic examination, sputum, BAL and NFS samples were inoculated into Eosin methylene blue agar, Brucella agar, blood agar, and chocolate agar media (Biomérieux, France), and incubated for 24-48 hours at 37°C. Colony identification was done using Vitek 2 Compact full automatic identification system (Biomérieux, France). In BAL cultures after 24-hour incubation, bacterial colonies with growth of 10^5^ and above were considered as pathogenic. The results were analyzed.

***Multiplex PCR/Reverse Line Blot Hybridization (M-PCR/RLBH) Analysis: ***The clinical samples underwent analyses including genomic DNA isolation, PCR step and reverse line blot hybridization on the assay were analyzed by the multiplex PCR method with CAP-Bac-PN Mix (Gen ID®; AutoimmunDiagnostika GmbH, Germany) kit. In this study, the detection of bacteria were done with RDB 2245/ RDB 2246 BAC HOSPITAL (Gen ID®; AutoimmunDiagnostika GmbH, Germany) multiplex PCR kit according to the manufacturer’s suggestions. The kit used in our study was able to detect DNA from ≥10^3^ bacteria.

***Statistical Analysis: ***SPSS version 21 software (IBM Corporation, NY, USA) was used for statistical analysis. Independent samples t-test was used for comparison of parametric data among groups, Mann-Whitney U test was used for comparison of nonparametric data, and x^2^ test was used for multiple non-parametric group comparisons. The limit for statistical significance was accepted as p<0.05.

## RESULTS

A total of 197 patients (117 male) were included in the study. The patients’ ages ranged from 4 to 88. The general characteristics of the patients are summarized in Table-I.

**Table-I:** General characteristics of the patients included in the study

	***n (%)***
Patient	197
Male	117(59.4)
Female	80(40.6)
*Diagnosis*	
CAP	147(74.6)
AECOPD	42(21.3)
Bronchiectasis	8(4.1)
*Sample*	
NFS	45(22.8)
Sputum	141(71.6)
BAL	11(5.6)
	Mean(±SD)
Age	40 (±23)
CRP	45.4 (±58)
Leucocyte	11.0 (±4.5)
Sedimentation	37 (±25)

CAP: community-acquired pneumonia,

AECOPD: acute exacerbation of chronic obstructive pulmonary disease,

NFS: Nasopharyngeal swabs, BAL: bronchoalveolar lavage, CRP:C-reactive protein

***Culture results: ***At least one bacterial isolate was detected in 62 (31.5%) patients’ culture. The most commonly grown bacteria based on the culture studies were *S. pneumoniae* (16.9%), *M. catarrhalis* (6.1%) and *S. aureus* (3.0%) (Table-II).

**Table-II:** The distribution of microorganisms isolated in the culture according to diagnosis

**n (%)**	**Total**	** CAP**	**AECOPD**	**Bronchiectasis**
No isolated	65 (33.0)	57(38.8)	6(14.3)	2(25.0)
Normal flora	70 (35.5)	48(32.7)	18(42.9)	4(50.0)
*S.pneumoniae*	32 (16.2)	23(15.6)	8(19.0)	1(12.5)
*M.catarrhalis*	12 (6.1)	8(5.4)	4(9.5)	-
*S. aureus*	6 (3.0)	5(3.4)	1(2.4)	-
*K. pneumoniae*	5 (2.5)	-	4(9.5)	1(12.5)
*E. coli*	5 (2.5)	4(2.7)	1(2.4)	-
*S.pneumoniae+ M.catarrhalis*	1 (0.5)	1(0.7)	-	-
*S.aureus+P.aeruginosa*	1 (0.5)	1(0.7)	-	-

CAP: community-acquired pneumonia, AECOPD: acute exacerbation of chronic obstructive pulmonary disease.

There were no significant correlations between patients’ culture isolate and gender or white blood cell count, C-reactive protein (CRP) and sedimentation values. However, there was a significant difference between age and culture growth (p = 0.033).

In terms of growth rate in culture, there was a significant difference between pediatric and adult patients (p = 0.026). Bacterial growth was detected in 7 of 41 pediatric patients and in 55 of 156 adult patients. The three most frequently detected bacteria in children *were S. pneumonia* (4), *E. coli* (2) and *M. catarrhalis *(1). The three most frequently detected bacteria in adult patients were *S. pneumoniae* (28), *M. catarrhalis* (11), and *S. aureus* (6). 

***PCR results: ***The detection rate of the pathogen was significantly higher in PCR method compared to the culture method (p <0.005). At least one pathogen was detected in 125 patients (63.5%).

The most frequently detected species by PCR were *S. pneumoniae* (32%) and *H. influenzae* (31%). The PCR-detected bacteria and their ratios classified according to diagnosis are given in Table-III.

**Table-III:** The distribution of the microorganisms detected by PCR according to diagnosis

**PCR results (n/%)**	**Total**	**CAP**	**AECOPD**	**Bronchiectasis**
Negative	72(36.5)	58(39.5)	13(31)	1(12.5)
*S.pneumoniae*	30(15.2)	23(15.6)	6(14.3)	1(12.5)
*S.pneumoniae+H.influensae*	29(14.7)	23(15.6)	6(14.3)	-
*H.influensae*	25(12.7)	15(10.2)	7(16.7)	3(37.5)
*M.pneumoniae*	5(2.5)	5(3.4)	-	-
*M.catarrhalis*	5(2.5)	2(1.4)	3(7.1)	-
*E.coli*	4(2)	3(2)	1(2.4)	-
*H.influenzaetipb +M.catarrhalis*	4(2)	3(2)	1(2.4)	-
*S.pneumoniae+M.catarrhalis*	4(2)	3(2)	-	1(12.5)
*S.aureus*	4(2)	4(2.7)	-	-
*H.influenzae tip b*	3(1.5)	1(0.7)	1(2.4)	1(12.5)
*H.influenzae+M.catarrhalis*	3(1.5)	3(2)	-	-
*H.influenzae+K.pneumoniae*	2(1)	-	1(2.4)	1(12.5)
*K.pneumoniae*	2(1)	-	1(4.8)	1(12.5)
*H.influenzaetipb+C.pneumoniae*	1(0.5)	1(0.7)	-	-
*H.influenzae tip b+S.aureus*	1(0.5)	-	1(2.4)	-
*H.influenzae+E.coli*	1(0.5)	1(0.7)	-	-
*H.influenzae+S.aureus*	1(0.5)	1(0.7)	-	-
*M.catarrhalis+S.aureus*	1(0.5)	1	-	-
Total	197	147	42	8

CAP: community-acquired pneumonia, AECOPD: acute exacerbation of chronic obstructive pulmonary disease.

When we compared the bacteria detection rate in PCR based on the specimen type, we found that the pathogen detection rate was highest in sputum (66.7%), followed by BAL (63.6%) and NFS (53.7%).There were no significant differences between detection of bacteria PCR and gender, white blood cell count, CRP and sedimentation values.

Also, when we compared the bacterial detection rates in PCR between children and adult patients we found a significant difference (p = 0.011). One or more pathogens were detected in 19 of 41 pediatric patients and in 106 of 156 adult patients. The most frequently detected bacteria in children were *S. pneumoniae* + *H. influenzae* (7), *S. pneumoniae* (5), *M. pneumoniae*(2) and *E. coli* (2). In adult patients, the three most commonly detected bacteria were *S. pneumoniae* (25), *H. influenzae* (24) and *S. pneumoniae* + *H. influenzae* (22).

***PCR and culture comparison results: ***When we compared the PCR and culture methods in terms of bacterial detection rate, the detection rate by PCR was significantly higher (p <0.005). While bacterial growth was detected in cultures from 62 patients, the PCR method was able to detect bacteria in 125 patients. The PCR method detected bacteria in 60 out of 62 patients with positive cultures. There were only two patients who had positive cultures but negative PCR.

In all patients who had *S. aureus* and *E. coli* in the culture, the same bacteria were detected in the PCR as well. In cases with culture positive for *S. pneumoniae*, *K. pneumoniae* and *M. catarrhalis*, PCR detected bacteria in all but one of these specimens. There was only one case in which culture and PCR detected different pathogens; the culture method grew *S. pneumoniae* while PCR detected *H. influenzae type b*.

When culture is considered to be the gold standard, the sensitivity of PCR method was 0.96, and the positive predictive value was found to be 0.95.There was a significant difference between the culture and PCR methods in terms of detection rates of multiple pathogens (P <0.001). Culture method was able to detect multiple bacteria in only two cases, while PCR detected multiple pathogens in 47 cases.

## DISCUSSION

Early identification of causative agents in LRTI, can reduce morbidity and prevent an overuse of antimicrobials. Conventional methods, such as culture and serology are not always adequate to detect lower respiratory tract pathogens. Therefore, new diagnosis methods are needed. So, we designed and performed this study to evaluate multiplex PCR and conventional culture method for their clinical efficacy. In our study, the rate of bacterial identification in clinical samples by PCR method was 63.5% while it was roughly half that in the culture method (31.5%).

It is well known that conventional culture tests have low sensitivity and specificity for detecting microorganisms.^5^^-^^7^ The other disadvantageous are in requirement of different culture media for different organisms and due to many variety of microorganisms the results are difficult to interpret. In the literature, many studies have reported higher pathogen identification rates with the PCR method.^11^^-^^16^ In a study conducted with pneumonia patients, microbial agents were detected in 39.1% of patients using the culture method while this ratio was 65.2% when using a PCR method.^16^ In another study conducted with BAL samples from 156 LRTI patients, investigators reported that the pathogen detection rate increased from 13% to 35% in *S. pneumoniae*, from 20% to 46% in *H. influenzae* and increased from 2 to 20 patients in detection of dual pathogen presence when using the PCR method compared to culture method.^17^ In another study investigating sputum samples from 76 children with pneumonia, the multiplex PCR method showed the presence of bacteria in 10 of 14 patients with initially negative culture results.^18^

In our study, there were only two cases with positive cultures in which PCR did not detect any pathogens. In addition, there was only one sample where culture and PCR detected different pathogens. Therefore, we suggest that multiplex PCR method is more reliable. Indeed, PCR sensitivity and specificity of 94-100% have been reported.^19^^,^^20^ In our study, the sensitivity of PCR method was 0.96 and the positive predictive value was calculated to be 0.95.

Another important finding of our study was that culture method detected only two multipl bacteria while PCR detected presence of multiple bacteria in 47 patients. *Wang et al.* has reported that they detected the presence of multiple bacteria in 35% of children under five years old with LRTI using PCR method.^21^ Moreover, *Lieberman et al*.^22^ reported detection of multiple pathogens in 35% of adult patients with LRTI. Another study conducted in Malaysia, reported that ratio as 17.7%.^16^ In our study, the multiple pathogen detection rate was 24% by PCR. The reasons for this might include inhibition of growth multiple species of bacteria due to the selectivity of culture conditions.

For rational antibiotic selection and treatment success, it is essential to know the frequency and resistance properties of microbiological agents in the general population prior to starting the experimental antibiotics regimen. Many national and regional studies on agents with culture and serological methods have been conducted and these studies have formed the foundation of empirical treatment. However, there are only a few studies with PCR. In our study, similar to previous studies, the most commonly detected bacteria in both culture and PCR were *Streptococcus pneumoniae, Haemophilus influenzae, Moraxella catarrhalis, Mycoplasma pneumoniae *and* Staphylococcus aureus.*

The multiplex PCR kit used in our study had the ability to detect atypical infectious agents such as *Mycoplasma pneumoniae, Chlamydia pneumoniae*and* Legionella pneumophila*. However, we did not examine these pathogens with culture and serological methods, which is one of the limitations of our study. There are difficulties in routine practice of culture and serological methods used in the diagnosis of atypical pathogens. The bacteria grow slowly (3-6 weeks) and with difficulty in bacterial growth media. Serological methods take 2-4 weeks and are therefore only useful for epidemiological studies.^23^ PCR methods have been reported to provide high sensitivity and specificity in simultaneous and rapid detection of such slow and hard growing atypical bacteria.^24^^,^^25^ In the literature, the detection range of atypical pathogens is quite wide (3-20%).^15^^,^^21^^,^^25^ In our study, only the PCR method was used for the detection of atypical agents, and *M. pneumoniae *was detected in 2.5% of the patients, *C. pneumoniae* in 0.5%, and *L. pneumophila* was not detected at all. Another limitation of our study was that we did not evaluate viral pathogens that are another common cause of LRTIs.

In conclusion, the multiplex PCR method is highly reliable and is superior in the detection of multiple pathogens and also provides rapid identification of bacteria and the etiological agents of infection. Therefore, we suggest that the widespread use of PCR methods will contribute to the success of LRTI treatments.

## Authors Contribution:

**O A:** Conception, design, analysis of samples, analysis and interpretation of data.

**YA:** Conception, design, analysis and interpretation of data, drafting and final approval of the manuscript.

**M O:** Analysis and interpretation of data.

## Notes

***Funding:*** None.
